# The antagonism between MCT-1 and p53 affects the tumorigenic outcomes

**DOI:** 10.1186/1476-4598-9-311

**Published:** 2010-12-07

**Authors:** Ravi Kasiappan, Hung-Ju Shih, Meng-Hsun Wu, ChikOn Choy, Tai-Du Lin, Linyi Chen, Hsin-Ling Hsu

**Affiliations:** 1 Division of Molecular and Genomic Medicine, National Health Research Institutes, 35 Keyan Road, Zhunan, Miaoli County 350, Taiwan; 2 Institute of Molecular Medicine, National Tsing Hua University, Hsinchu 300, Taiwan

## Abstract

**Background:**

MCT-1 oncoprotein accelerates p53 protein degradation via a proteosome pathway. Synergistic promotion of the xenograft tumorigenicity has been demonstrated in circumstance of p53 loss alongside MCT-1 overexpression. However, the molecular regulation between MCT-1 and p53 in tumor development remains ambiguous. We speculate that MCT-1 may counteract p53 through the diverse mechanisms that determine the tumorigenic outcomes.

**Results:**

MCT-1 has now identified as a novel target gene of p53 transcriptional regulation. MCT-1 promoter region contains the response elements reactive with wild-type p53 but not mutant p53. Functional p53 suppresses MCT-1 promoter activity and MCT-1 mRNA stability. In a negative feedback regulation, constitutively expressed MCT-1 decreases p53 promoter function and p53 mRNA stability. The apoptotic events are also significantly prevented by oncogenic MCT-1 in a p53-dependent or a p53-independent fashion, according to the genotoxic mechanism. Moreover, oncogenic MCT-1 promotes the tumorigenicity in mice xenografts of p53-null and p53-positive lung cancer cells. In support of the tumor growth are irrepressible by p53 reactivation *in vivo*, the inhibitors of p53 (MDM2, Pirh2, and Cop1) are constantly stimulated by MCT-1 oncoprotein.

**Conclusions:**

The oppositions between MCT-1 and p53 are firstly confirmed at multistage processes that include transcription control, mRNA metabolism, and protein expression. MCT-1 oncogenicity can overcome p53 function that persistently advances the tumor development.

## Background

Mutations or loss of the tumor suppressor p53 gene have been documented in more than 50% of human cancers [1-3]. Functional p53 is involved in the regulation of genomic integrity, growth arrest, DNA repair, programmed cell death, and cell differentiation [3-5]. As a transcription factor, p53 binds specifically to the consensus DNA sequence consisting of two copies of the 10-bp motif 5'-RRRC(A/T)(T/A)GYYY-3', in which R is a purine and Y is a pyrimidine, separating by a 1-13 base pair (bp) junction [6-8]. These specific sequences are recognized in the p53 regulatory genes, such as Pirh2 [9], Cop1 [10], Waf-1/p21 [11], MDM2 [12], Bax [13], and PCNA [14]. Numerous p53 downstream targets are implicated in tumor suppression. But Pirh2, MDM2, and Cop1 are ubiquitin ligases implicated in tumor development that mediate p53 degradation in a proteosome manner [9,10,15]. The genome-wide ChIP studies have also indentified the p53-regulatory genes BCL2A1, PTK2 and VIM that associate with tumor formation [16,17].

The activity of p53 exerts paradoxically anti-apoptotic and pro-survival effects, which are essential for the development of an organism and may turn p53 into a tumor promoter. As a comprehensive guardian of genome integrity, p53 confers the survival-promoting advantages of cancer cells [18]. More substantial evidence have emerged that p53 protects cells from the genotoxin-induced apoptosis [19-21]. Though p53 induces Bax activation and apoptosis, relocating the p53 protein to mitochondria does not trigger tumor cell death, conversely grants apoptotic resistance to ionizing radiation [22]. Moreover, p53 reduces the oxidation-induced DNA damage and apoptosis [23-25]. Overall, p53 has its dark side that enhances the cell surviving mechanism and potentially inititates tumorigenicity. Exploration of p53 antagonists or p53 downstream targets which are implicated in tumorigenesis, is thus a very important task.

MCT-1 (multiple copies in T cell malignancy 1) oncogene is highly expressed in the human lymphomas [26,27]. Overexpression of MCT-1 promotes cell survival, proliferation, checkpoint bypass, and anchorage-independent growth [26,28,29]. Constitutively expressed MCT-1 transforms normal breast epithelial MCF-10A cells [30], and increases the tumorigenicity of breast cancer MCF-7 cell xenografted mice, possibly through promoting angiogenesis and anti-apoptosis [31]. MCT-1 protein interacts with the ribosome and associates with the cap complex by the putative RNA-binding motif, PUA domain [32,33]. Ectopic MCT-1 also promotes translational initiation of many cancer-related mRNAs, including BCL2L2, Cyclin D1, TFDP1, MRE11A and E2F1 [34]. Furthermore, ectopically expressed MCT-1 decreases p53 mRNA levels and p53 protein stability *in vitro *[35,36].

The regulations in opposition between p53 and MCT-1 have now been verified *in vitro *and *in vivo*. The wild-type p53 targeting the *MCT-1 *gene promoter could affect the presentation of MCT-1 mRAN and protein. Reciprocally, MCT-1 depresses *p53 *gene promoter, mRNA stability, and protein function. Moreover, the reactivation of p53 cannot restrain the MCT-1 tumorigenic impacts on H1299 (p53 null) lung cancer cells xenografted mice and the stimulation of p53 repressors (MDM2, Pirh2, and Cop1). As well, the oncogenic MCT-1 persistently promotes the xenograft tumorigenicity of A549 (p53 wild-type) lung cancer cells. These data reveal that MCT-1 advances cellular malignancy and tumorigenic potency independent of p53 status.

## Results

### MCT-1 gene and protein are downregulated by p53

**Figure 1:**
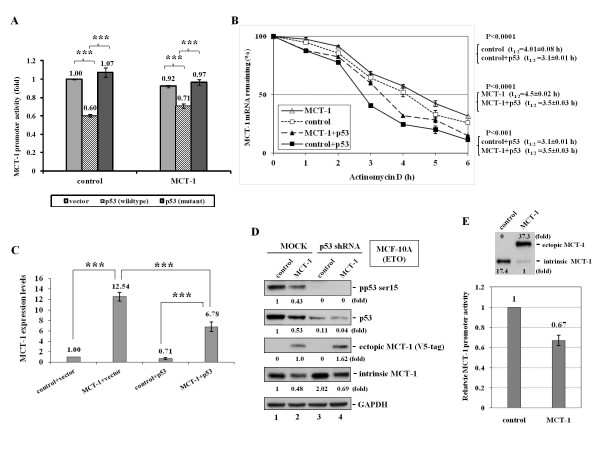
**MCT-1 gene and protein expressions are decreased by p53**. The pGL3-MCT-1 promoter construct was transiently transfected into H1299 (p53 null) cells. (A) Reduced luciferase activity of the pGL3-MCT-1 reporter was identified when H1299 cells (control) expressed the wild-type p53 but not the mutant p53. Similar effects were recognized in context of MCT-1 overexpression (MCT-1) (B) The half-life (t_1/2_) of MCT-1 mRNA was analyzed by qRT-PCR after actinomycin D (5 μg/ml) treatment for the indicated time. Quantitative data was acquired as normalized with 18S rRNA levels. Cellular MCT-1 mRNA turnover was greater induced by p53 expression (control + p53) than p53 null condition (control). The exogenic MCT-1 mRNA decayed quickly in p53 renovation (MCT-1 + p53) versus (vs.) without p53 expression (MCT-1). (C) As determined with qRT-PCR analysis, the p53 knock-in H1299 moderately depressed intrinsic MCT-1 mRNA levels (control vs. control + p53). As well, the exogenic MCT-1 mRNA levels were significantly depressed by p53 (MCT-1 vs. MCT-1 + p53). (D) MCF-10A cells were under ETO genotoxic stress for 4 h. The ectopic (V5-tag) and intrinsic MCT-1 protein in MCF-10A (p53-proficient) cells were reversely elevated because *p53 *gene silence (p53 shRNA) relative to MOCK experiment. (E) Ectopic expression of MCT-1 reduced pGL3-MCT-1 promoter activity together with decrease in intrinsic MCT-1 protein. The overall cellular MCT-1 protein amounts (intrinsic plus ectopic) were more than a 2.2-fold elevation after MCT-1 induction. Statistics were analyzed with Student's t test. ***p < 0.0001.

To investigate whether p53 affected intrinsic *MCT-1 *gene activation, the non-small cell lung cancer H1299 (p53 null) cells were transfected with pCDNA vector alone or pCDNA-p53 to rebuild p53 function (control + p53, MCT-1 + p53). For examining the MCT-1 promoter function, a 1.3 kb *MCT-1 *gene promoter segment (-1301 to +37) was engineered into a pGL3-luciferase basic vector. The pGL3-MCT-1 promoter construct was then introduced into the control H1299 cells (control) that expressed with the vector alone (pCDNA), the wild-type p53 (pCDNA-p53), or the mutant p53 (pCDNA-p53mt135) (Figure 1A). Cells presenting wild-type p53, the pGL3-MCT-1 luciferase activity was depressed markedly to 60% of promoter activity of cells without p53 (vector) (p < 0.0001). Conversely, the pGL3-MCT-1 promoter activity was unchanged by the mutant p53, revealing that only the functional p53 particularly deactivated the *MCT-1 *gene promoter. Similar data was also presented in context of pLXSN-MCT-1 expressed H1299 (MCT-1) that only wild-type p53 affected MCT-1 promoter activity. To exclude the possibility that p53 non-specifically obstructed the promoter function, the 5'LTR promoter segment of pLXSN was constructed into a pGL3-Luciferase basic vector. Obviously, the p53 showed no effect on the reporter activity of pGL3-5'LTR (Additional File 1A).

To further analyze the half-life (t_1/2_) of MCT-1 mRNA in H1299 cell present or absent of p53, Actinomycin D was used for inhibition of *de novo *gene transcription (Figure 1B). Cellular RNA samples were then harvested at the indicated intervals to measure MCT-1 mRNA quantities by qRT-PCR study. Results indicated that the intrinsic MCT-1 mRNA decayed faster in cells having p53 (t_1/2 _= 3.1 h) (control + p53, filled square) than those lacking p53 (t_1/2 _= 4.01 h) (control, open square). As well, the *MCT-1 *gene expression was examined after cells transfecting with pLXSN vector alone (control) or pLXSN-MCT-1 (MCT-1). Similar to intrinsic MCT-1 mRNA turnover, p53 renovation also negatively regulated the steady-state of ectopic MCT-1 mRNA that decomposed rapidly (t_1/2 _= 3.5 h) (MCT-1 + p53, filled triangle) comparing with p53 absent condition (t_1/2 _= 4.5 h) (MCT-1, open triangle). The effect of p53-dependent destabilization of MCT-1 mRNA stability was inhibited as MCT-1 mRNA was much stable in the MCT-1 + p53 cells (t_1/2 _= 3.5) than the control + p53 cells (t_1/2 _= 3.1), possibly ectopic MCT-1 performing a negative impact on p53 role.

By quantifying the overall MCT-1 mRNA levels with qRT-PCR analysis (Figure 1C), MCT-1 mRNA levels in the *p53 *gene-restored H1299 cells (control + p53) were found to be decreased to 71% of that of sample without the p53 expression (control + vector). Consistent with decrease in cellular MCT-1 mRNA levels by p53, the levels of exogenic MCT-1 mRNA (MCT-1 + p53) (6.78) also dramatically reduced to 54% of that of the vector controls (MCT-1 + vector) (12.54) as well. These data demonstrate that the p53 reactivation can effectively repress *MCT-1 *gene presentation.

Further analysis was examined whether p53 reduction conversely improved MCT-1 expression in normal breast epithelial MCF-10A cells with wild-type p53 and regular genetic background (Figure 1D). MCT-1 protein levels were detected by the specific Ab against a synthetic peptide (a.a. 72-88) of MCT-1 polypeptide. Following etoposide (ETO) genotoxin treatment for 4 h, cellular p53 was accumulated and functionally activated. MCT-1 amount was found to be a 2-fold increase after p53 silencing (p53 shRNA) relative to the non-silence control (MOCK) (lanes 1 vs. 3). Moreover, ectopic MCT-1 protein (V5-tag) was recognized by the V5-epitope Ab that showed a 1.62-fold elevation after p53 knockdown comparative to the non-silence group (lanes 2 vs. 4). These validate that p53 presence actually counteracts MCT-1 protein production.

The unexpected data indicated that intrinsic MCT-1 protein was dramatically inhibited after ectopic MCT-1 expression (Figure 1E). As well, the autoregulation of *MCT-1 *gene presentation was identified as the pGL3-MCT-1 promoter activity was diminished by ectopic MCT-1 to 67% of that of control group. Even so, the entire MCT-1 protein amounts (ectopic plus intrinsic) still promoted to a 2.2-fold induction as compared with control group. These data establish for the first time that MCT-1 controls itself, via a feedback loop involving the promoter downregualtion.

### Interaction of p53 with the MCT-1 promoter region

**Figure 2:**
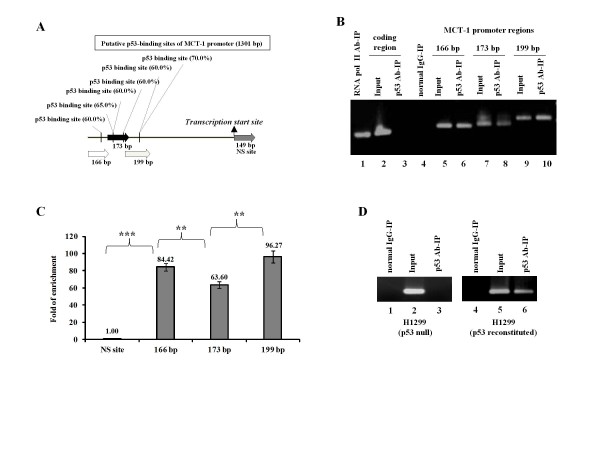
**MCT-1 promoter associates with p53**. (A) Six putative p53-binding sites on the MCT-1 promoter region are illustrated. The primers for ChIP assays are indicated by 166, 173 and 199 bp on the MCT-1 promoter region with the horizontal arrows. (B) Following the genotoxic stress, MCF-10A genomic DNA was immunoprecipitated (IP) with p53 and RNA polymerase II Ab. The original input and the Ab-IP complexes were amplified by a conventional PCR analysis with the primers for MCT-1 promoter, MCT-1 coding region, or *GAPDH *gene. MCT-1 promoter DNA was identified by primers specific for the 166, 173, and 199 bp fragments in the IP complex of p53 Ab (lanes 6, 8, 10) but not normal IgG (lane 4). MCT-1 coding region was undetectable in the p53-IP complex (lane 3). The *GAPDH *gene interacting with RNA polymerase II was recognized as a positive control (lane 1). (C) In quantitative PCR (q-PCR) comparison, the relative values were normalized with the original input and then compared with the non-specific MCT-1 coding region (NS site). (D) MCT-1 promoter DNA fragment (166 bp) was detected only in the p53-IP complex of the p53-restored H1299 cells under the genotoxic stress. Statistical analysis was done with the Student's t test. **p < 0.002; ***p < 0.0001.

MCT-1 promoter region was searching for the consensus p53-binding element, 5'-RRRC(A/T)(T/A)GYYY-3', using the NTI vector program. Six potential p53-binding sites were identified at the MCT-1 promoter region that located between nucleotides (nt.) -1301 and -801 (Figure 2A). ChIP analysis was studied whether the MCT-1 promoter DNA associated with the activated p53 protein under ETO genotoxic stress in MCF-10A cells (Figure 2B). Immune complex of p53 antibody (p53 Ab-IP) were PCR-amplified with the primers (Additional File 2) for the MCT-1 promoter region that correspondingly produced DNA fragment sizes of 166, 173 and 199 base pair (bp). Conversely, the nonspecific (NS) site located at the MCT-1 coding region (nt. 1~149) was undetectable in the p53 Ab-IP complex. The RNA polymerase II specifically recognized *GAPDH *gene as a positive control, but no DNA associated with normal IgG. Using a qPCR analysis to quantify ChIP results, the 166 and 199 bp locations exhibited higher associations with p53 than that of the 173 bp region (p < 0.002) (Figure 2C), indicating their differential connections with p53 protein. Furthermore, the parental (p53 null) or the *p53 *gene restored H1299 cells (p53-reconstituted) were conducted with ChIP studies (Figure 2D). The MCT-1 promoter DNA was only detectable in the p53-IP complex of the p53-reconstituted sample but not in the parental group. Though no p53 repressor element (p53TRE) is noticed in the MCT-1 promoter region, the relation of p53 protein with MCT-1 promoter may obstruct *MCT-1 *gene transcription.

**Figure 3:**
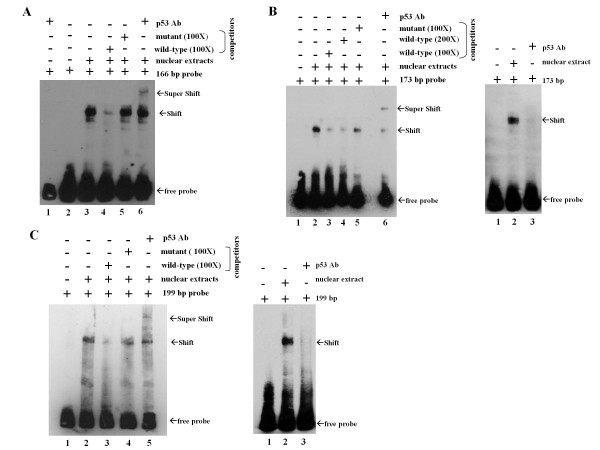
**Binding of p53 to the MCT-1 promoter region**. EMSA was conducted by incubating the MCT-1 promoter probes with nuclear extracts of MCF-10A cells after the genotoxin (ETO) treatment. (A) The biotin-labeled 166 bp probe formed a complex with the nuclear protein (lane 3). This complex was partially depleted by the wild-type (lane 4) but not by the mutant (lane 5) p53-responsive elements in a 100X excess concentration. The presence of p53 in complex was confirmed by inducing a super-shift complex with p53 Ab (lane 6). No specific interaction between the probe and p53 Ab was recognized (lane 1). (B) The biotin-labeled 173 bp probe formed a DNA-protein complex with the nuclear protein (lane 2). This complex was partially reduced by the wild-type competitor at concentrations of 100X excess (lane 3) and 200X excess (lane 4), but it was not depleted by the mutant competitor at a concentration of 100X excess (lane 5). The presence of p53 in complex was confirmed by the p53 Ab inducing a super-mobility shift (lane 6). No specific complex formed between the probe and p53 Ab (right panel). (C) The biotin-labeled 199 bp probe interacted with the nuclear protein (lane 2). This DNA-protein complex was specifically interrupted by wild-type (lane 3) but not the mutant p53-responsive competitor at a concentration of 100X excess (lane 4). A super-shift band was identified as p53 Ab reacted with the DNA-protein complex (lane 5). Again, no particular complex produced among the probe and p53 Ab (right panel).

Electrophoretic-mobility shift assay (EMSA) was investigated whether p53 protein closely interacted with the MCT-1 promoter sites. Biotin-labeled probes (166, 173, and 199 bp), covering the promoter region from -1301 to -801 (Figure 2A), were prepared and incubated with nuclear extracts (NE) from MCF-10A cells after etoposide (ETO) treatment that stimulated and stabilized p53 protein. The biotinylated 166 bp probe formed a complex with the nuclear protein as indicated by a mobility shift (Figure 3A, lane 3). This specific DNA-protein complex was dramatically competed by a 100-fold excess of the p53 consensus oligonucleotides (lane 4) but not the mutant p53-responsive element (lane 5). Furthermore, p53 existence in the complex was proved by generating a super-shifted band after incubation with the p53-specific antibody (lane 6). But, no obvious complex formed between the p53 Ab and the DNA probe alone (lane 1).

On the other hand, the biotinylated 173 bp probe revealed a specific interaction with the nuclear protein (Figure 3B, lane 2), which was greatly attenuated by adding a 100- or 200-fold excess of p53 consensus oligonucleotide (lanes 3 and 4). A super-shifted band was evidently induced by the p53 Ab, confirming the presence of p53 protein in the complex (lane 6). On the contrary, the DNA-protein complex was unable to be depleted by the mutant p53-responsive element (lane 5). As well, no detectable complex was produced between the p53 Ab and the DNA probe alone (Figure 3B, right panel).

Another DNA-protein complex was recognized while the nuclear extracts reacting with the biotinylated 199 bp probe (Figure 3C, lane 2). This complex was also significantly repressed by a 100-fold excess of the wild-type p53 consensus oligonucleotide (lane 3), but it was insignificantly contended by the mutant p53-responsive element (lane 4). A super-shifted mobility caused by the p53 Ab clarified p53 being in the DNA-protein complex (lane 5). Moreover, no specific DNA-protein complex was generated between p53 Ab and the DNA probe (Figure 3C, right panel). These data have evidently proved that p53 protein interacts with the promoter of *MCT-1 *gene.

### MCT-1 inhibits p53 promoter activation and protein expression

**Figure 4:**
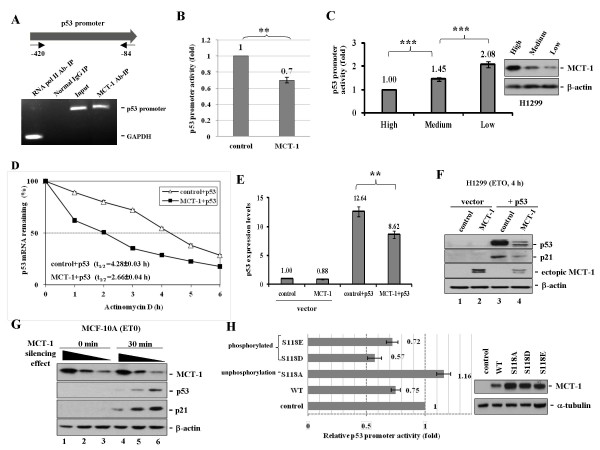
**MCT-1 decreases p53 promoter function and protein expression**. (A) The primers of p53 promoter region (-420 to -84) were hybridized with the ChIP complex. The p53 promoter DNA specifically contained in the IP complex of MCT-1 Ab but not normal IgG. (B) Luciferase activity of the pGL3-p53 promoter was depressed by ectopic MCT-1 (MCT-1) compared with control H1299 (control). (C) The pGL3-p53 promoter was conversely activated while MCT-1 silencing in H1299 context. (D) The half-life of exogenic p53 mRNA was decreased in ectopic MCT-1 group (MCT-1 + p53) relative to the corresponding control (control + p53) (P < 0.0001). (E) The overall p53 mRNA productions in the p53-restored H1299 cells were declined evidently in ectopic MCT-1 background (MCT-1 + p53). (F) Upon etoposide (ETO) exposure for 4 h, ectopic MCT-1 reduced the p53 functional activation of p21 (lanes 3 vs. 4). (G) The shRNA interference of intrinsic MCT-1 in MCF-10A cells conversely stimulated p53 and p21 proteins upon ETO treatment for 30 min. (H) The effects of wild-type MCT-1 (WT) and MCT-1 mutants that were directly mutated on Serine 118 residue (S118) to Alanine (S118A), Aspartic acid (S118D), and Glutamic acid (S118E) were studied. The dephosphorylation mutant (S118A) significantly improved the pGL3-p53 promoter function, but pGL3-p53 promoter was still repressed by cells expressing the phosphorylation-mimetic MCT-1 (S118D and S118E) and wild-type MCT-1. Statistical data was assessed with the Student's t test. **p < 0.002; ***p < 0.0001.

ChIP assay was again evaluated if the MCT-1 protein reciprocally associated with *p53 *gene promoter (Figure 4A). The p53 promoter-specific primers ranging from -420 to -84 were identified whether the *p53 *promoter DNA contained in the IP complex of MCT-1 Ab. In a PCR amplification analysis, approximately 8.9% of *p53 *promoter DNA was discovered in MCT-1 immune complex (IP). Control experiments had identified that *GAPDH *gene specifically associated with RNA polymerase II, but no genomic DNA was coupled with a normal IgG. Different from p53 specific binding with the MCT-1 promoter DNA (Figure 3), no interaction was detected between the p53 promoter DNA and MCT-1 protein in the EMSA study (data not shown). It is possible that MCT-1 could cooperate with other undisclosed molecule(s) that closely interact with the *p53 *gene promoter.

The proximal p53 promoter region (-188 to +12) contains a full promoter activity in the response to DNA damage [37,38]. To study whether ectopic MCT-1 antagonized p53 promoter activity, the p53 promoter DNA (-188 to +23) segment was cloned into the pGL3-Luc basic vector and then transfected into H1299 cells. The luciferase activity of pGL3-p53 promoter in ectopic MCT-1 group was decreased approximately to 70% of the respective control group (control) (Figure 4B), indicating that MCT-1 functionally inactivated p53 promoter. The activity of CMV promoter of pCDNA3.1 that was cloned into the pGL3-Luc basic vector was not affected by the MCT-1, excluding the possibility that ectopic MCT-1 non-specifically affected the promoter function (Additional File 1B).

To analyze if MCT-1 status directly involved in *p53 *gene deactivation, *MCT-1 *gene was knocked down by MCTS1 shRNA in H1299 cells that reduced cellular MCT-1 protein to different degrees (high, medium, low) (Figure 4C). By contrast with suppression of MCT-1 protein levels, the pGL3-p53 promoter activity was progressively improved. As compared with low p53 promoter activity in high MCT-1 context, the reporter activity was conversely elevated up to a 2-fold induction while MCT-1 declined significantly. These verify that MCT-1 plays a critical role in regulation of *p53 *gene promoter.

We speculated that MCT-1 also repressed the *p53 *gene reactivation in H1299 cells, the metabolism of exogenic p53 mRNA was analyzed after *de novo *gene transcription being inhibited by actinomycin D (Figure 4D). The remaining of p53 mRNA quantity was assessed with qRT-PCR analysis at each time point. Unlike exogenic p53 mRNA decayed quickly under MCT-1 oncogenic influence (t_1/2 _= 2.66 h) (MCT-1 + p53, close square), the p53 mRNA was relatively stable in control group (t_1/2 _= 4.28 h) (control + p53, open triangle). Further assessment with qRT-PCR study, we found that the total p53 mRNA quantities in ectopic MCT-1 cells (MCT-1 + p53) were dropped to 68% of that of controls (control + p53) (Figure 4E). Taken together, these results firstly illustrate that MCT-1 inhibits the overall p53 mRNA expression by decreasing p53 promoter function and p53 mRNA stability.

Consistent with the *p53 *gene deactivation and p53 mRNA reduction (Figure 4B-4E), the ectopic p53 that functionally stimulated the p21 protein expression upon etoposide (ETO) exposure for 4 h was greatly suppressed by MCT-1 (MCT-1 + p53) relative to the control H1299 (control + p53) (Figure 4F, lanes 3 vs. 4). Confirmative data also revealed that MCTS1 shRNA interference reversely elevated p53 and p21 proteins in MCF-10A cells exposure to ETO (30 min) (Figure 4G). Thus, MCT-1 overexpression effectively counteracts the p53-p21 signaling function that is reversible after MCT-1 knockdown.

MCT-1 overexpression activating the extracellular signal-regulated kinase activity (ERK) is link with p53 degradation (35). To test the functional domain responsible for p53 promoter deactivation (Figure 4H), the Serine 118 (S118) residue of the potential MAPK kinase phosphorylation site on MCT-1 protein was specifically mutated to Alanine (S118A). As well, MCT-1 (S118) residue was modified to Glutamic acid (S118E) and to Aspartic acid (S118D) that were mimetic to the phosphor-S118 MCT-1. Surprisingly, only the S118A mutant restored the p53 promoter activity significantly, but the phosphorylation-mimetic MCT-1 (S118E and S118D) still decreased p53 promoter to an extent comparable to wild-type (WT). These suggest that the serine 118 residue on MCT-1 is essential and sufficient for inhibition of p53 promoter function.

### MCT-1 oncogenic effects in p53-dependent and p53-independent manners

**Figure 5:**
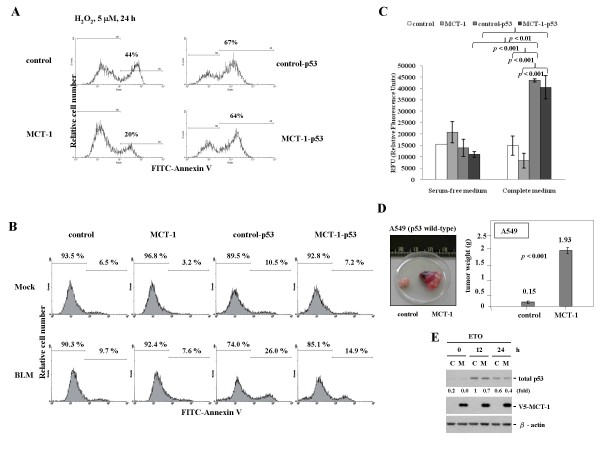
**MCT-1 oncogenicity in p53-dependent and p53-independent manners**. (A) Apoptotic results were analyzed with Annexin V staining. MCF-10A cells containing p53 (control and MCT-1) or silencing p53 (control-p53 and MCT-1p53) were exposed to H_2_O_2 _for 24 h. MCT-1 prevented cell death in a p53-dependent manner. (B) Apoptotic events were insignificant in p53 proficient cohorts (control and MCT-1) as cells treated with BLM for 24 h. At p53 reduced condition, ectopic MCT-1 decreased apoptotic effect (MCT-1-p53), contrasting with high apoptotic events in control group (control-p53). (C) Fluorimetric Cell Migration Array Kit was analyzed cell movement ability induced by p53 deficiency but unaffected by MCT-1 status. (D) The A549 (p53 wild-type) lung cancer cells expressed MCT-1 oncoprotein still demonstrated with higher tumor burdens than control xenografts. (E) Oncogenic MCT-1 slightly reduced p53 accumulation after etoposide (ETO) treatment.

To evaluate the cooperative impact of p53 loss and MCT-1 oncogenicity on cell survival (Figure 5A), MCF-10A cells induced with (MCT-1) or without MCT-1 (control) were subsequently knocked down cellular p53 protein (control-p53 and MCT-1-p53). Following exposure to hydrogen peroxide (H_2_O_2_, 5 μM) for 24 h, cell death was analyzed with FITC-Annexin V flow cytometry. Ectopic MCT-1 cells showed a lower apoptotic outcome (20%) than that was detected in p53-proficient controls (44%). After p53 loss, cell death induced by the oxidative damage was promoted equally in control-p53 (67%) and MCT-1-p53 (64%) situations. These indicate that MCT-1 protects cells from the oxidative stress relying on p53 function.

On the other hand, a radiomimetic agent, Bleomycin (BLM) was treated MCF-10A cells (Figure 5B). Death events were not significantly induced in p53 proficient background (control and MCT-1). But, cells deficient with p53 (control-p53 and MCT-1-p53) increased incidence of apoptosis upon BLM exposure. Even so, the apoptotic populations were still less detected in MCT-1-p53 group (14.9%) than control-p53 set (26.0%), revealing that MCT-1 protected cells from BLM genotoxicity independent of p53 function.

Cell migratory ability was moreover investigated by Fluorimetric cell migration assay (Figure 5C). While culturing in the complete media, cell mobility was notably enhanced in p53 knockdown (control-p53 and MCT-1-p53) but unaffected by inducing MCT-1. Cell motility mainly enhanced by loss of p53 function may be related with Rho signaling pathway that controls actin cytoskeleton organization as literatures demonstrate [39].

To confirm MCT-1 tumorigenic potency in a p53 wild-type background, A549 lung adenocarcinoma cells ectopically expressed MCT-1 oncogene also slightly suppressed p53 accumulation in the response to ETO genotoxin (Figure 5E). Following A549 cells subcutaneously injected into the nude mice, the tumorigenic results had evidently proved that MCT-1 oncogene strongly promoted the tumor development to a 12.8-fold increase in comparison with the control A549 xenografts (Figure 5D). Therefore, MCT-1 oncogenicity could go beyond p53 function in the tumorigenic development.

### MCT-1 promotes tumorigenicity independent of p53 status

It was unidentified whether wild-type *p53 *gene reconstitution in the p53 null background relieved MCT-1 oncogenicity. The impact of p53 restoration on chromosome copy number was surveyed by a cytogenetic study [36]. The intrinsic gene mutations (chromosome amplification) in H1299 background (copy number = 101) did not obviously improve after *p53 *gene reconstitution (copy number = 97) or change as MCT-1 overexpressed (copy number = 97). These suggest that p53 reactivation probably cannot alter MCT-1 tumorigenic outcomes.

**Figure 6:**
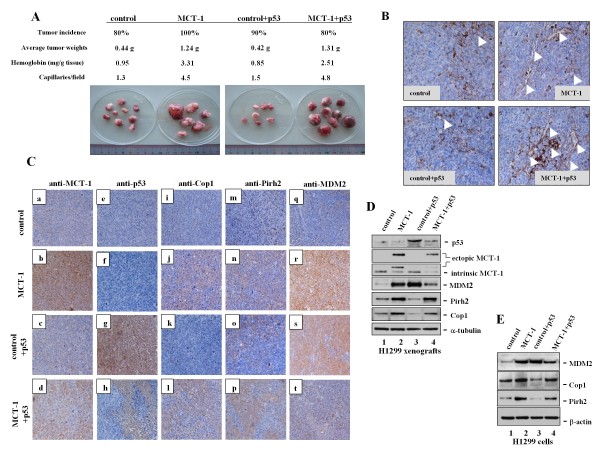
**Reactivation of p53 cannot prevent MCT-1 tumorigenicity**. (A) Different types of H1299 cancer cells (control, MCT-1, control + p53, MCT-1 + p53) were inoculated into the athymic nude mice. As indicated with CD31 immunostaining analysis, the vascular index (capillaries/field) was calculated by six randomly selected fields from each tumor biopsy. Tumor weights, hemoglobin amounts, and capillary densities were promoted in the MCT-1 and MCT-1 + p53 xenografts. (B) Immunohistology study of the tumors with CD31 Ab revealed a higher density of microvessels (indicated with arrows) in the MCT-1 and MCT-1 + p53 xenografts than those identified in the control and control + p53 xenografts. (C) Immunohistology data indicated the proteins (brownish) and H&E counterstaining (blue) in the tumors. The basal levels of MCT-1 protein expressed in control and control + p53 samples (a, c). MCT-1 proteins were intensively enhanced in the ectopic MCT-1 tumors (b), whereas the MCT-1 + p53 tumors reduced in MCT-1 concentration (d). The control and MCT-1 tumors were p53 null (e, f). Lower p53 levels were noticed in the MCT-1 + p53 tumors (h) than that of the control + p53 xenografts (g). Cop1, Pirh2 and MDM2 were all enriched in the MCT-1 tumors (j, n, l, p, r). MDM2 was also enriched in the control + p53 xenografts (s), but it was dramatically decreased in the MCT-1 + p53 xenografts (t). (D) The p53 protein was reduced by ectopic MCT-1 in the tumors (lanes 3 vs. 4). Similarly, intrinsic and ectopic MCT-1 quantities were all decreased by p53 restoration (lanes 2 vs. 4). MDM2 levels were promoted after MCT-1 increment (lanes 1 vs. 2) or p53 reactivation (lanes 1 vs. 3). But, MDM2 was comparatively reduced in the concomitant increase of p53 and MCT-1 (MCT-1 + p53) (lanes 3 vs. 4). (E) Consistent with the tumor results, H1299 cells expressing MCT-1 significantly improved MDM2 protein levels (lanes 1 vs. 2), but MDM2 was rather decreased in MCT-1 + p53 sample than control + p53 group. Pirh2 and Cop1 amounts were persistently enriched in the MCT-1 and MCT-1 + p53 backgrounds.

To explore whether MCT-1 oncogene continually antagonizes p53 function *in vivo*, different types of H1299 cells (control, MCT-1, control + p53, MCT-1 + p53) were subcutaneously inoculated into athymic BALB/c mice. The xenograft tumor burdens were enhanced dramatically in the mice engrafted with MCT-1 and MCT-1 + p53 expressed cells as compared with their corresponding control and control + p53 cells (p < 0.0001) (Figure 6A). Moreover, the MCT-1 xenografts produced drastically larger tumors containing with higher hemoglobin levels (p < 0.0001) and vascular counts (p < 0.0001) than those were identified in the control and control + p53 xenografts. Though p53 functionally activated p21 expression (Figure 4F), the wild-type *p53 *gene transfer still unsuccessfully repressed tumor growth in ectopic MCT-1 background that concomitantly increased in micro-vascularization as evaluated with the endothelial marker CD31 immunohistology staining (Figure 6B). As a result, the tumorigenicity and angiogenecity are not suppressed by p53 renovation; probably due to p53 only function effectively in the early tumor initiation stage. Once the tumors have developed, p53 activation disables to repair the genetic mutations and control the tumor growth.

The interrelation between p53 and MCT-1 in the tumors were subsequently verified by immunohistochemistry study (Figure 6C, a-t). Consistent with *in vitro *cellular results, MCT-1 was also decreased markedly in the tumors with p53 expression (MCT-1 + p53) (d). However, MCT-1 was produced highly in the tumors without p53 (MCT-1) (b). Though p53 was greatly restored in the H1299 background (control + p53) (g), it was still comparatively reduced because ectopic MCT-1 induction (MCT-1 + p53) (h). Intriguingly, the p53 suppressors, Cop1 and Pirh2, were stimulated predominantly in ectopic MCT-1 background (j, n, l, p). Although MDM2 amounts were significantly enhanced either by ectopic MCT-1 (MCT-1) (r) or by p53 restoration (control + p53) (s), the p53-mediated MDM2 induction in tumors was declined strikingly while simultaneously expressing MCT-1 and p53 (MCT-1 + p53) (t).

These protein expressions were furthermore inspected in the tumors (Figure 6D). Due to p53 influence, lower intrinsic and ectopic MCT-1 levels were detected in MCT-1 + p53 tumors than in MCT-1 tumors (lanes 2 vs. 4). On the other hand, less p53 quantities observed in the MCT-1 + p53 tumors than in control + p53 ones (lanes 3 vs. 4). These indicate that MCT-1 works against p53 in the tumor development. Consistent with the findings in MCF-10A (Figure 1E), the auto-regulation of MCT-1 was manifestly detected *in vivo *because endogenic MCT-1 decreased in quantity as ectopic MCT-1 expressed (lanes 1 vs. 2).

MDM2 oncogene is trans-activated by p53, functioning as an E3 ubiquitin ligase to promote p53 degradation [40]. Our results had shown that MDM2 quantities were remarkably elevated in the MCT-1 xenografts as compared with the control H1299 xenografts (lanes 1 vs. 2). Even though MDM2 was promoted in the p53 renovation (control + p53) (lanes 1 vs. 3) (Figure 6D), the extent of MDM2 induced by p53 became insignificant when exogenic MCT-1 suppressed p53 action on MDM2 stimulation (lanes 3 vs. 4). As well, ectopic MCT-1 intensified Pirh2 and Cop1 productions that potentially destabilized p53 protein (lanes 1 vs. 2). Likewise, the appearances of MDM2, Pirh2, and Cop1 in the H1299 cellular context were equivalent with their manifestations in tumors (Figure 6E). Stimulations of MDM2, Pirh2, and Cop1 could explain how oncogenic MCT-1 attenuates p53 protein accumulation and tumor-suppressive role during tumor development as the literatures report [41-43].

**Figure 7:**
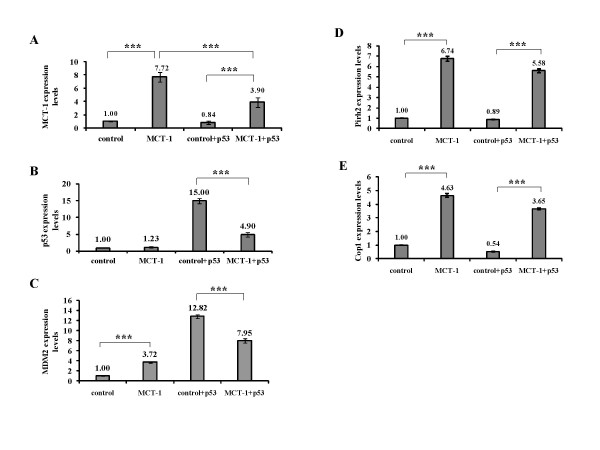
**MCT-1 deactivates p53 but activates p53 inhibitors in tumor development**. H1299 tumor biopsies were subjected to qRT-PCR analysis. (A) MCT-1 mRNA levels in the MCT-1 + p53 xenograft tumors were decreased comparative to the MCT-1 xenografts. (B) The p53 mRNA levels in the tumors were significantly declined in the MCT-1 + p53 xenografts relative to the control + p53 xenografts. (C) MDM2 mRNA amounts were moderately increased by ectopic MCT-1 and further promoted by p53 restoration. Low MDM2 mRNA levels were presented that was probably due to ectopic MCT-1 depress the p53 function. (D-E) Pirh2 and Cop1 mRNA levels were constitutively enhanced by oncogenic MCT-1 in a p53-independent manner. Statistical analysis was performed with the Student's t test. ***p < 0.0001.

Supportive evidence was acquired from qRT-PCR analysis as well. MCT-1 mRNA levels in H1299 xenograft tumors were considerably decreased by p53 reactivation (Figure 7A) (MCT-1 vs. MCT-1 + p53, p < 0.0001). On the other hand, when the *p53 *gene was transferred into H1299 cells, p53 transcripts were dramatically suppressed by ectopic MCT-1 (Figure 7B) (control + p53 vs. MCT-1 + p53, p < 0.0001). Unlike *p53 *gene deactivation, MDM2 mRNA levels were somewhat induced by ectopic MCT-1 (Figure 7C) (control vs. MCT-1, p < 0.0001). Though MDM2 transcripts were promoted in a p53-dependent mode, this consequence apparently decreased when MCT-1 inhibited p53 function (control + p53 vs. MCT-1 + p53, p < 0.0001). Differently, the transcripts of *Pirh2 *and *Cop1 *genes constitutively promoted by ectopic MCT-1 were only moderately affected by p53 status (Figure 7D and 7E). Upregulation of *MDM2*, *Pirh2*, and *Cop1 *gene molecules provide other lines of evidence that MCT-1 downregulates p53 in tumorigenic process at the transcriptional stage.

In support of the tumorigenic outcomes, the phosphor-activation of AKT and MAPK were found to be enhanced in MCT-1 + p53 xenograft tumors relative to the other cohorts (control, control + p53, MCT-1) (Additional File 3A). Additionally, the integrin-β4 was enriched particularly in MCT-1 + p53 xenograft tumors. Furthermore, the molecules involving in the oncogenic potential, H-Ras and HIF-1α mRNA levels, all showed approximately 1.6-fold increases in MCT-1 + p53 xenografts compared with those in the control + p53 xenografts (Additional File 3B). Stimulations of these anti-apoptotic molecules, which can enhance cancer cell proliferation and survival mechanisms, emphasize that the p53 reactivation under MCT-1 oncogenic stress fails to slow down the tumor development.

## Discussion

### The antagonism between MCT-1 oncogene and p53 tumor suppressor

MCT-1 oncogene is a dangerous foe to p53 function, playing multiple roles in promoting chromosome instability and tumor growth [36]. Constitutively activating MCT-1 decreases p53 protein via a proteasome pathway [35]. We now demonstrate that MCT-1 reduces p53 mRNA levels accompanied with *p53 *gene promoter inactivation and p53 mRNA destabilization, which well correspond to inhibit p53-p21 pathway (Figure 4B-4F). Conversely, *MCT-1 *gene interference stimulates *p53 *gene promoter and improves p53-p21 expression (Figure 4C and 4G). Moreover, the original results have also demonstrated that p53 reciprocally interacts with the *MCT-1 *gene promoter that potentially suppresses MCT-1 oncogenicity in a feedback mechanism (Figure 2B-2D and Figure 3).

The mutual counteractions between p53 and MCT-1 at the gene and protein stages are comparable to the negative regulation between MDM2 and p53 [44,45]. As well, the reciprocated transcriptional repression between MCT-1 and p53 resembles the mechanism of Twist oncogenic activity that obstructs the p53 tumor-suppressive function [46]. Another intriguing finding is that the induction of MCT-1 exerts a self-directed inhibition on intrinsic MCT-1 protein (Figure 1D-1E, and 6D), by which the overall MCT-1 protein levels can be systematically controlled using an autonomous regulation in its promoter function (Figure 1E). The integral self-control could determine the steady state of MCT-1 activity that may critically regulate cell growth or tissue homeostasis. Similar effect has been identified that Myc overexpression contributes to tumorigenesis and myc expression is controlled through an autoregulatory circuit in non-transformed cells, by which elevated Myc protein amounts lead to down-regulation of myc transcription [47]. As well, overexpression of c-myc gene leads to a significant decrease in endogenous N-myc levels [48].

### Reactivation of p53 cannot suppress MCT-1 tumor-promoting effect

The tumor promotion in MCT-1 expressed H1299 xenografts represents the synergistic consequences of p53 null and MCT-1 induction (Figure 6). Our data have demonstrated that p53 reactivation cannot compromise the tumorigenic results induced by MCT-1 oncogene. MCT-1 + p53 xenograft mice thus develop significantly larger tumors with higher hemoglobin levels and micro-vessel density than the other xenograft tumors (control, MCT-1, control + p53). We have previously shown that *p53 *gene add-in cannot rescue the p53-deficient cells from MCT-1 oncogenic impact on genome destabilization, but actually increases incidence of aneuploidy from 42.8% to 95% [36]. Thus, p53 renovation fails to inhibit MCT-1-induced aneuploidization that could predispose to many carcinogenic endpoints as the documents report [49]. Another important fact is that MCT-1 oncogene confers cellular resistance to the oxidative pressure depending on p53 function (Figure 5A). In the other way, the genotoxn-induced cytotoxicity is reduced particularly when p53 is abrogated in ectopic MCT-1 cells (Figure 5B). But independent of MCT-1 function, cell migratory ability is promoted predominantly by p53 deficiency (Figure 5C), which could be coupled with the signaling activation of Rho pathway [39]. Regardless of p53 function, the tumor-promoting consequence is still largely promoted in A549 (p53 wild-type) lung cancer cells with a constitutive activation of MCT-1 (Figure 5D), further revealing that MCT-1 oncogenicity could overcome p53 action in the tumor development.

### The p53 inhibitors are stimulated in MCT-1 oncogenic background

MCT-1 synergistically induces the oncogenic molecules in the p53 deficient background, which could be important for cell malignant transformation (Additional file 3). The biochemical and genetic information all support that ectopic MCT-1 induces tumor promotion accompanied with the enrichment of MDM2, Pirh2 and Cop1 in the tumors (Figure 6C-6D and 7C-7E), moreover explaining why p53 knock-in cannot suppress MCT-1 tumorigenic effects. In a p53-dependent manner, escalating p21 and MDM2 levels have been demonstrated that p53 restoration indeed actively regulates its downstream targets (Figure 4F and 6D-6E). Likewise, the reactive p53 capably depresses MCT-1 protein in the MCT-1 + p53 xenograft tumors (Figure 6C-6D). Though MDM2 stimulated by p53 reconstitution, the MCT-1 inhibitory impact on p53 expression is also reflective to decline *MDM2 *gene and protein in MCT-1 + p53 xenografts (Figure 6C-6D, 7C). Furthermore, MCT-1 dramatically enhances *Pirh2 *and *Cop1 *gene expressions that could eventually contribute to p53 destabilization (Figure 7D-7E). Overall, MCT-1 oncogenicity competently surpasses p53 tumor-suppressive ability that abolition of MCT-1 tumorigenic strength through gain of p53 function becomes impossible.

Ectopic MCT-1 enhances p53 negative regulators that can decline p53 function in tumor prevention *in vivo*. These negative influences as well reflect to upregulate MCT-1 promoter, MCT-1 mRNA stability, and overall MCT-1 mRNA amounts *in vitro*. MCT-1 promoter function reduced by wild-type p53 shows somewhat improvement in cells expressing MCT-1 (0.71) relative to control group (0.6) (Figure 1A), potentially because ectopic MCT-1 reduces the p53 depressing effect. In evaluation of MCT-1 mRNA turnover (Figure 1B), the p53-dependent suppression on MCT-1 mRNA stabilization is reduced by ectopic MCT-1 that MCT-1 mRNA is more stable in MCT-1 + p53 sample (t_1/2 _= 3.5) than control + p53 group (t_1/2 _= 3.1) (p < 0.001). Besides, the overall MCT-1 mRNA levels in ectopic MCT-1 sample are much more enhanced (6.78) than control group (0.71) (Figure 1C). This is partly due to ectopic MCT-1 stimulate p53 inhibitors that can cancel the p53-dependent suppression on MCT-1 transcription.

Genetic evidence has implicated that α6β4 integrin signaling in promoting tumor angiogenesis and invasion [50]. These can be enhanced by HIF-1α and Ras up-expression as well [51-54]. A selective enhancement of pro-survival molecules (Integrin-β4, p-AKT, p-MAPK, H-RAS and HIF-1α) under MCT-1 oncogenic stress could increase cancer cell growth and angiogenecity that are substantially advantageous for tumor development (Additional file 3). The MDM2-p53 pathway has been recognized as an ideal therapeutic target for cancer treatment [55]. MCT-1 promotes angiogenesis that might be achieved by deregulating p53 downstream targets, such as, TSP1, VEGF, and COX-2 [56-58]; by inhibiting the MDM2-HIF-1α interaction [59,60]; or by enhancing the Twist-HIF-1α regulation [53]. Understanding of the crosstalk between MCT-1 and p53 in depth may facilitate the development of a new promising cancer therapeutic strategy that improves the therapeutic efficacy.

How do MCT-1 and p53 counteract each other? MCT-1 modulates p53 degradation through the extracellular signal-regulated kinase activity (ERK) because the ERK antagonist effectively restores p53-p21 expression [35]. In a negative feedback loop, p53 may deactivate ERK function to change MCT-1 stability [27]. Our important novel findings indicate that the S118A mutant of MCT-1 fails to inhibit p53 promoter activity but that is still affected by the mimetic ERK-phosphorylated MCT-1 (S118E and S118D) (Figure 4H). For that reason, MCT-1 could regulate the *p53 *gene promoter involving ERK pathway, or direct interaction with ERK molecule [27,35]. In addition, p53 regulates and represses RNA poly III transcription activity that may control MCT-1 protein synthesis or the oncogenic effects on cell growth as the literatures indicate [61,62]. MCT-1 may also functionally resemble E6 and MDM2 oncoproteins, releasing RNA poly III from repression by p53 that highly enhances pol III transcription activity for protein synthesis, cell growth, and malignant phenotypes [63,64].

## Conclusions

Our results uncover an important reciprocated regulation between MCT-1 oncogene and p53 tumor suppressor. Achieving a counterbalance between them may determine tumor prevention or development. The wild-type *p53 *gene reactivation is not capable to suppress the tumor growth promoted by MCT-1. Thus, *MCT-1 *gene knockout or dysfunction of MCT-1 activity could be another significant stratagem for inhibition of the tumorigenicity.

## Methods

### Antibodies and reagents

Antibodies (Abs) against the following proteins were purchased from different sources as indicated: p53, p21, Cop1, Pirh2, integrin-β4 and AKT (Santa Cruz Biotechnology, Santa Cruz, CA); MDM2, α-tubulin, GAPDH and β-actin (Abcam, Cambridge, UK); phospho-MAPK (Thr^202^/Tyr^204^), phospho-AKT (Ser^473^), phospho-p53 (Ser^15^) and MAPK (Cell Signaling Technology, Danvers, MA); and CD31 (BD Pharmingen, San Diego, CA). The V5-epitope Ab (Invitrogen, Carlsbad, CA) identified the ectopically expressed V5-tagged MCT-1. The MCT-1 rabbit antibody (Zymed Laboratories Inc, San Francisco, CA) for detecting intrinsic MCT-1 was generated against a synthetic peptide (a.a. 72-88). Actinomycin-D and etoposide were acquired from Sigma (St. Louis, MO). pCMV-p53 and pCMV-p53mt135 plasmid DNA were obtained from Clontech Laboratories Inc. (Mountain View, CA).

### Cell culture and transfections

Non-small cell lung cancer cells, H1299 (p53 null) were co-transfected with pLXSN/MCT-1-V5 and pCDNA3.1/p53. Another lung cancer cell line A549 (p53 will-type) was transfected with pLXSN vector alone or pLXSN/MCT-1-V5. The stable master cultures were established and maintained as previously described [35]. Normal breast epithelial MCF-10A cells were transfected with pLXSN or pLXSN/MCT-1-V5 that subsequently transfected with pMKO.1 puro p53 short hairpin RNA2 (shRNA2) or a mock vector as previously described [36]. The *MCT-1 *gene was abrogated in parental H1299 or MCF-10A cells by transfection with the pGeneClip MCTS1 shRNA vector (SA Biosciences Corp, Frederick, MD), using jetPEI™transfection reagent (Polyplus-transfection, New York, NY). These stable transfectants were cultured with the medium containing puromycin (0.5 μg/ml).

### Quantitative real-time polymerase chain reaction (qRT-PCR)

Total RNA was extracted from cells or mouse tumor tissues using TRIzol reagent (Invitrogen). The cDNA was synthesized from 2 μg RNA using Oligo (dT)_12-18 _primer and Superscript II reverse transcriptase (Invitrogen). MCT-1 mRNA levels were measured as previously described [36]. The specific primers for *p53, MDM2, Cop1, Pirh2, HIF-1α *and *H-Ras *genes were designed by Primer Express software to ensure a single 72, 99, 123, 137, 76 and 69-bp amplicon. These probes were labeled NFQ (quencher) and FAM (reporter) and synthesized by Integrated DNA Technologies (Applied Biosystems, Foster City, CA). Reactions were performed in a 20 μl reaction mixture containing 150 ng cDNA, 10 μl TaqMan PCR Master Mix (Applied Biosystems) and 1 μl corresponding TaqMan probe. Reactions were run on the ABI Prism 7900 Fast Real-Time PCR system in triplicate as follows: 95°C for 10 min, 45 cycles of a 15-second denaturing at 95°C and 1 min annealing at 60°C. The mRNA levels were calculated. Cycle threshold (ΔCt) = Ct target gene - Ct endogenous control (18S rRNA gene).

### Plasmid construction

MCT-1 promoter DNA was isolated from the MCF-10A genome by PCR amplification using the forward primer, 5'-GAGCGGTACCAGGTTTTTAAATTTTT-3' (-1301 to -1284), and the reverse primer, 5'-GGAAGCTTTTAGGCAACCGG-3' (+37 to +25). The PCR products were cloned into Kpn*I *and Hind*III *restriction sites of pGL3-Luciferase basic vector (pGL3-MCT-1 promoter). The p53 promoter segment (-188 to +23) was amplified by PCR using the forward primer, 5'-CGAGCTCGTCGGCGAGAATCCTGACT-3' (-188 to -170), and the reverse primer, 5'-GGAAGCTTGGACGGTGGCTCTAGACTTT-3' (+3 to +23). The PCR products were constructed in the pGL3-Luciferase basic vector with Sac*I *and Hind*III *restriction sites (pGL3-p53 promoter). The 5'LTR promoter of the pLXSN vector was PCR-amplified with the primers 5'-GGGGTACCTAGACCACTCTACCCTATTC-3' and 5'-CCAAGCTTACACCCTAACTGACACACAT-3' to respectively generate Kpn*I *and Hind*III *sites at the 5'-and 3'-ends of the DNA fragment. The amplified 5'LTR promoter was cloned into the pGL3-Luciferase basic vector using Kpn*I *and Hind*III *sites (pGL3-5'LTR).

The CMV promoter was removed from pCDNA3.1 (+)/hygro vector using Mlu*I *and Nhe*I *and inserted into the pGL3-Luciferase basic vector with the same restriction sites to generate the CMV reporter construct (pGL3-CMV).

### Site-directed mutagenesis on MCT-1

Three PCR primer sets were designed to generate the mutant strands of MCT-1 on Serine 118 residue (S118). The primer set for S118A included the forward primer 5'-GTCCAGGCTTAACTGCTCCTGGAGCTAAG-3' and the reverse primer 5'-CTTAGCTCCAGGAGCAGTTAAGCCTGGAC-3'. The primer set for S118D included the forward primer 5'-CATGTGTCCAGGCTTAACTGACCCTGGAGCTAAGCTTTAC-3' and the reverse primer 5'-GTAAAGCTTAGCTCCAGGGTCAGTTAAGCCTGGACACATG-3'. The primer set for S118E included the forward primer 5'-CATGTGTCCAGGCTTAACTGAGCCTGGAGCTAAGCTTTAC-3' and the reverse primer 5'-GTAAAGCTTAGCTCCAGGCTCAGTTAAGCCTGGACACATG-3'. Following the manufacturer's protocol for the GeneEditor™*in vitro *site-directed mutagenesis system (Promega, Madison, WI), the insertion of wild-type MCT-1 constructed on pGEX-5X-1 plasmid was used as the mutagenesis template. The plasmid DNA was denatured, phosphorylated, annealed with the mutagenic oligonucleotides, and incubated with T4 DNA polymerase and T4 DNA ligase (Promega) at 37°C for 90 min. Mutant plasmids were transformed into BMH 71-18 mutS competent cells and selected with the GeneEditor™antibiotic selection mix, and subsequently transformed into high-efficiency JM109 competent cells followed by the selection of the ampicillin and GeneEditor™antibiotic selection mix. For long term storage, the mutants were transformed into the DH5α competent cells.

Wild-type MCT-1 cDNA was digested from pLXSN-MCT-1 plasmid by EcoR*I *& Xho*I*. Point mutant inserts were digested from pGEX-5X-1-MCT-1 mutant plasmid by EcoR*I *and BamH*I*. Inserts were amplified by pfu DNA polymerase (Stratagene, La Jolla, CA) using F-MCT1-Hpa (forward primer 5'-CCCGTTAACGCCACCATGTTCAAGAAATTTGATGAAAAAGAAAATGTG-3') and R-MCT1-Cla (reverse primer 5'-CCCATCGATTTTATTTCAGTTATCTAATTTGCGGCCGCTTTATATGTCTTCATATG CCACAGCCCATC-3'). PCR products were digested with Hpa*I *and Cla*I *before constructing into pLHCX vector (BD Biosciences, Palo Alto, CA). The recombinant plasmids were transformed into OneShot MachI T1 cells (Invitrogen), followed by colony PCR, enzyme digestion, and sequencing analysis. Three copies of FLAG Tag was PCR-priming from pCMV3Tag8 vector (Stratagene) with the FLAG Tag primer set, i.e. forward primer 5'-ATTTGCGGCCGCACTCGAGGATTACAAGGAT-3' and reverse primer 5'-TAAAGCGGCCGCCTATTTATCGTCATC-3'. The PCR product of three copies of Flag Tag was cloned into the Not*I *site of pLHCX-MCT-1 plasmid. MOCK control (pLHCX vector alone) and FLAG-tagged pLHCX-MCT-1 (wild-type and mutants) plasmids were individually transfected into PT67 packaging cell line (BD Biosciences) using a Lipofectamine agent (Invitrogen). Transfectants were selected by 100 μg/ml hygromycin (MD Bio, Taipei, Taiwan). The viral supernatants were collected in a hygromycin-free medium and then infected MCF-10A cells (ATCC, Manassas, VA).

### Luciferase activity assay

To analyze the luciferase activity, 0.5 μg reporter plasmid (pGL3-MCT-1 promoter, pGL3-p53 promoter, pGL3-5'LTR, or pGL3-CMV), 0.1 μg β-galactosidase plasmid, and 1 μl JetPEI reagent were mixed with 75 mM NaCl solution for 30 min before transfection into H1299 or MCF-10A cells. After 48 h, the cells were washed with 1X PBS and incubated with 200 μl lysis buffer (Promega) for 30 min at -80°C. Cell extracts (70 μl) were added into 96-well microtiter plates and combined with 30 μl luciferase assay reagent (Promega). The reaction was detected with a Hidex Chameleon machine, and then analyzed with Mikro Win2000 software. To analyze β-galactosidase activity, 30 μl lysates were incubated with 22 μl 1X ONPG, 47 mM sodium phosphate, and 1 mM MgCl_2 _solution. Reactions were incubated at 37°C for 10 min and measured with the spectrophotometer at OD 420 nm.

### Chromatin immunoprecipitation (ChIP) assay

ChIP experiments were performed according to the manufacturer's protocol (Upstate Biotechnology, Lake Placid, NY). MCF-10A (p53 proficient) or p53-restored H1299 (2 × 10^7^) cells were exposed to 40 μM etoposide for 4 h, fixed with 1% formaldehyde for 10 min at room temperature, neutralized with 125 mM glycine for 5 min, washed twice with PBS, and the cells were scraped off with PBS containing the protease inhibitor cocktail. Cell pellets were suspended in a 400 μl SDS lysis buffer (1% SDS, 10 mM EDTA, 50 mM Tris-HCl, pH 8.0, protease inhibitor cocktail) and incubated on ice for 15 min followed by shearing of the genomic DNA into 200-1000 bp fragments by a sonicator (Bioruptor UCD-200). After cleaning the insoluble materials by centrifugation, supernatants were diluted with a 900 μl ChIP dilution buffer (0.01% SDS, 1% Triton X-100, 1.2 mM EDTA, 167 mM NaCl, 16.7 mM Tris-HCl, pH 8.1, protease inhibitor cocktail). Samples were pre-cleared with a 60 μl salmon sperm DNA/Protein G agarose slurry for 1 h at 4°C. An aliquot (10 μl) of the supernatants were kept as input materials, and the remaining samples (990 μl) were respectively incubated with 2 μg p53 Ab, 2 μg MCT-1 Ab, 1 μg RNA polymerase II Ab (positive control), or 1 μg normal mouse IgG (negative control) for 24 h at 4°C. The protein-DNA immune complexes were incubated with 60 μl salmon sperm DNA/Protein G agarose slurry for 1 h at 4°C. Beads were washed sequentially with the low salt buffer (20 mM Tris-HCl, pH 8.0, 2 mM EDTA, 150 mM NaCl, 0.1% SDS, 1% Triton X-100), the high salt buffer (20 mM Tris-HCl, pH 8.0, 2 mM EDTA, 500 mM NaCl, 0.1% SDS, 1% Triton X-100), the LiCl buffer (250 mM LiCl, 1% IGEPAL-CA630, 1 mM EDTA, 10 mM Tris-HCl, pH 8.0, 1% deoxycholic acid), and then rinsed twice with TE buffer (10 mM Tris-HCl, pH 8.0, 1 mM EDTA). Afterward, the protein-DNA complexes were eluted from the beads with 1% SDS (200 μl) at room temperature for 15 min. To reverse the cross-linked protein-DNA complexes, samples were diluted to 50 mM NaCl followed by incubation with 10 μg RNaseA and 10 μg proteinase K at 65°C for 24 h. The eluted DNA was purified with a PCR Purification Kit (Qiagen, Valencia, CA) and subjected to the conventional PCR and q-PCR. Conventional PCR products were resolved on a 2% agarose gel and stained with ethidium bromide. For q-PCR analysis, we employed the SYBR green system using the ABI Prism 7900 Fast Real-Time PCR system and determined the threshold cycle numbers (Ct). All the relative Ct values were normalized to the inputs and compared between samples.

### Electrophoretic-mobility shift assay (EMSA)

EMSA was conducted with a Gel-Shift kit according to the manufacturer's protocol (Panomics, Fremont, CA). The nuclear extracts were prepared after MCF-10A (2 × 10^7^) cells were exposed to 40 μM etoposide (ETO) for 4 h. The biotin-labeled MCT-1 promoter probes (166, 173 and 199 bp) corresponding to the nucleotides -1301 to -1135, -1142 to -969, and -1000 to -801 on the promoter regions were PCR amplified by forward and reverse primers as listed (Additional File 2). The PCR-amplified DNA probes were clarified by gel extraction kit (Qiagen). Nuclear extracts (5 μg) were pre-incubated with 1X EMSA binding buffer and 1 μg poly d(I-C) for 5 min at room temperature followed by incubation with 30 ng of biotin-labeled MCT-1 probe at 15°C for 30 min. The competition experiments were performed by including a 100- or 200-fold excess of unlabeled wild-type or mutant p53 consensus sequences in the reactions for 20 min prior to incubation with the biotin-labeled probe. For the super-shift assay, 1 μg of p53 antibody (SC-126 X) (Santa Cruz) was pre-incubated with the reaction for 1 h prior to adding the probe. Protein-DNA complexes were resolved with 6% non-denaturing polyacrylamide gel in 0.5X Tris-borate/EDTA buffer (100 mM Tris, 90 mM boric acid, 1 mM EDTA) at 4°C and transferred to an Immobilon positively-charged nylon membrane (Millipore, Billerica, MA) for 1 h at 300 mA. The transferred oligonucleotides were immobilized by UV crosslinking for 3 min. The membranes were reacted with the blocking buffer followed by reaction with Streptavidin-HRP and development with ECL reagent.

### Cell apoptotic analysis

To evaluate apoptotic cell death, MCF-10A cells were treated with 5 μM H_2_O_2 _or 40 mU Bleomycin for 24 h followed by staining with a Annexin V apoptosis detection kit (BD Biosciences) for 15 min. Afterward, apoptotic cells were evaluated by BD FACS Calibur Flow Cytometry (Becton-Dickinson, San Jose, CA).

### Cell migration assay

MCF-10A cells were essayed for migratory ability with QCM™24-Well Fluorimetric Cell Migration Array Kit (Chemicon International Inc., Temecula, CA). Cells (5 × 10^5 ^cells) were seeded in the culture chamber with an 8 μm pore size polycarbonate membrane. Five hundred microliter of serum-free or the complete DMEM/F-12 medium was added to the lower chamber. After incubating for 16 h at 37°C in a CO_2 _incubator, the non-migratory cells were carefully removed and the chamber membranes were inserted into a fresh well with 225 μl pre-warmed Cell Detachment Solution for 30 min in a 37°C incubator to detach cells, followed by adding 75 μl Lysis Buffer/CyQuant GR^® ^dye solution for 15 min at room temperature. Reaction mixtures (200 μl) were added into a 96-well micro-titer plate for detection of fluorescence absorbance at excitation/emission filter sets 485/530 nm using a Hidex Plate Chameleon (SisLab, Milano, Italy) apparatus.

### Tumorigenicity, hemoglobin assay, and immunohistochemistry studies

Eight-week-old female BALB/c nude mice (BALB/cAnN-Foxn1nu/CrlNarl) were injected with H1299 cancer cells (control, MCT-1, control + p53, MCT-1 + p53). This animal experiment was approved by Animal Use Protocol in National Health Research Institutes (NHRI-IACUC-096049-A). Each mouse was inoculated with 2 × 10^6 ^cells suspended in 100 μl RPMI medium at both subcutaneous sites. When tumor sizes had reached approximately 4-6 mm, the tumors were resected and weighed. The portions of tumor tissues were processed for hemoglobin levels, immunoblotting, qRT-PCR, and immunohistochemistry analysis as previously described [36].

## List of abbreviations

MCT-1: multiple copies in T-cell malignancy 1; bp: base pair; mRNA: messenger RNA; shRNA: short hairpin RNA; NFQ: non-fluorescent quencher; FAM: 5-carboxyfluorescein; PBS: phosphate-buffered saline; HRP: horseradish peroxidase; ChIP: chromatin immunoprecipitation; EMSA: electrophoretic mobility shift assay; qRT-PCR: quantitative real-time polymerase chain reaction; Luc: firefly luciferase; Ab: antibody; min: minute; H&E: hematoxylin and eosin; ECL: enhanced chemiluminescence; ETO: etoposide.

## Competing interests

The authors declare that they have no competing interests

## Authors' contributions

RK performed q-RT-PCR, CHIP, EMSA, cloning, luciferase, Western blotting, animal experiments, and sketched the manuscript. HJS performed gene silencing, DNA cloning, Western blotting, and cell migration analysis. MHW performed the cell cycle profiling study. COC performed apoptotic analysis. TDL helped with luciferase reporter assays in MCT-1 mutants. LC did immunohistochemistry study. HLH supported and supervised the entire project, interpreted data, and approved publication after critically revising the manuscript. All authors have read and approved the final paper.

## Supplementary Material

Additional file 1 **Luciferase activity of the pGL3-5'LTR (pLXSN) promoter and the pGL3-CMV (pCDNA3.1) promoter in H1299 cells**. (A) There were no significant changes of 5'LTR promoter (pLXSN vector) activity in the presence or absence of p53. (B) Ectopic expression of MCT-1 did not affect CMV (pCDNA vector) promoter activity in either p53-null or p53-positive conditions. Click here for file

Additional file 2 **Sequences of the primers, the probes, and the competitors**. Lists of primer sequences used for ChIP and EMSA assays, as well the nucleotide sequence of wild-type and the site-specific mutant of p53-responsive elements used for the EMSA competition assays. Click here for file

Additional file 3 **Oncogenic molecules are promoted in the MCT-1 xenograft tumors**. (A) The significant elevations of integrin-β4, p-AKT, and p-MAPK proteins were particularly recognizable in the MCT-1 + p53 tumors, whereas these proteins were rather reduced in other types of xenograft tumors (control, MCT-1, and control + p53). (B) The expressions of *H-Ras *and *HIF-1α *genes, potentially relating to cell malignancy, were promoting in the MCT-1 + p53 xenograft tumors. Click here for file
