# Supplementary figures and images for: The antagonism between MCT-1 and p53 affects the tumorigenic outcomes
Source: Mol Cancer. 2010 Dec 7;9:311. doi: 10.1186/1476-4598-9-311 (PMC3019166; doi:10.1186/1476-4598-9-311)

## Additional file 1

**A**

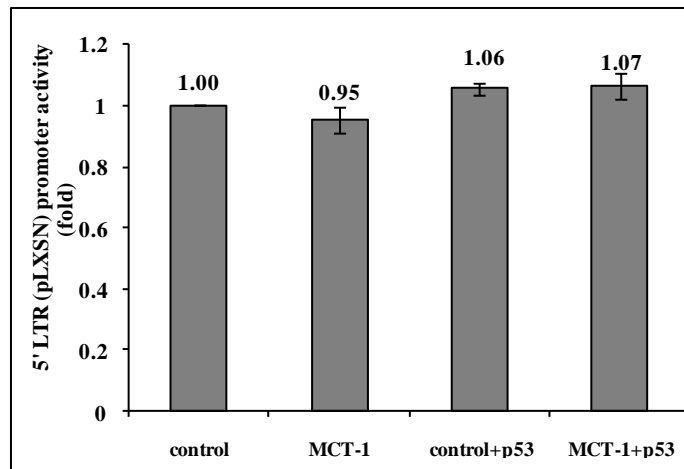

**B**

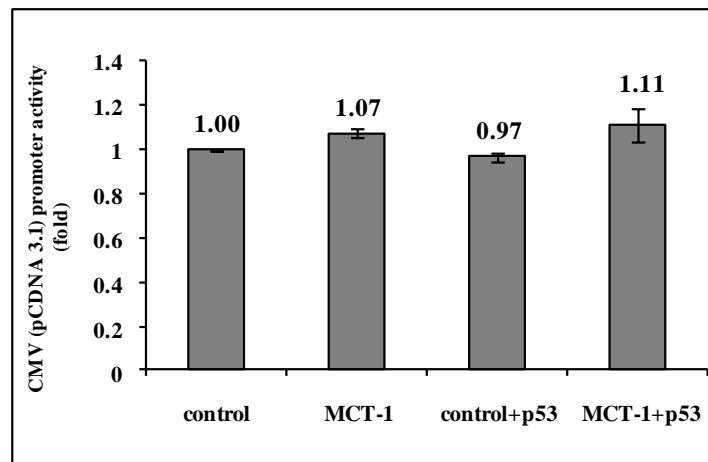

Supplement: Additional file 1 — Luciferase activity of the pGL3-5'LTR (pLXSN) promoter and the pGL3-CMV (pCDNA3.1) promoter in H1299 cells. (A) There were no significant changes of 5'LTR promoter (pLXSN vector) activity in the presence or absence of p53. (B) Ectopic expression of MCT-1 did not affect CMV (pCDNA vector) promoter activity in either p53-null or p53-positive conditions. [file 1476-4598-9-311-S1.PDF]

Additional file 3

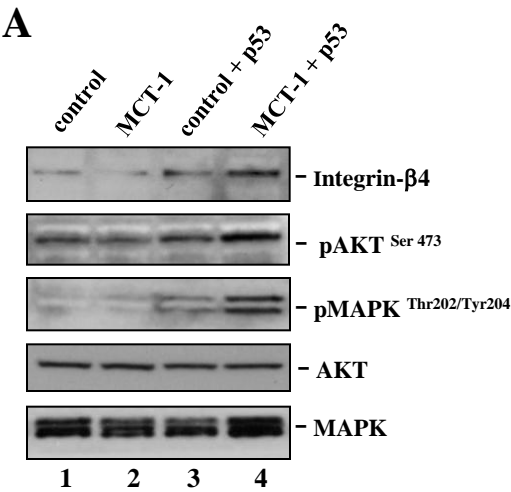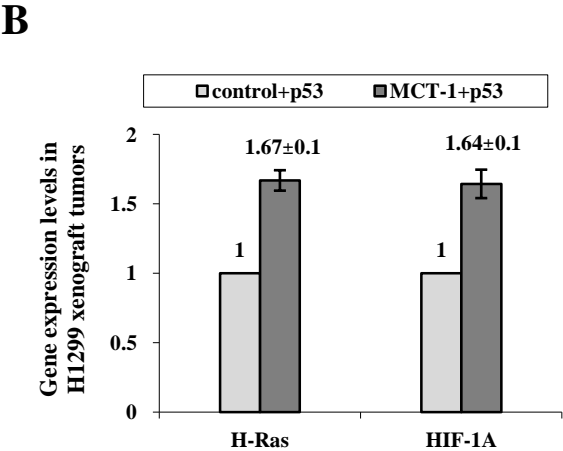

Supplement: Additional file 3 — Oncogenic molecules are promoted in the MCT-1 xenograft tumors. (A) The significant elevations of integrin-β4, p-AKT, and p-MAPK proteins were particularly recognizable in the MCT-1 + p53 tumors, whereas these proteins were rather reduced in other types of xenograft tumors (control, MCT-1, and control + p53). (B) The expressions of H-Ras and HIF-1α genes, potentially relating to cell malignancy, were promoting in the MCT-1 + p53 xenograft tumors. [file 1476-4598-9-311-S3.PDF]
