# Supplementary material for: The antagonism between MCT-1 and p53 affects the tumorigenic outcomes
Source: Mol Cancer. 2010 Dec 7;9:311. doi: 10.1186/1476-4598-9-311 (PMC3019166; doi:10.1186/1476-4598-9-311)
Supplement: Additional file 2 — Sequences of the primers, the probes, and the competitors. Lists of primer sequences used for ChIP and EMSA assays, as well the nucleotide sequence of wild-type and the site-specific mutant of p53-responsive elements used for the EMSA competition assays. [file 1476-4598-9-311-S2.PDF]

Additional file 2

Samples	Nucleotide sequences
**166 bp (-1301~ -1135)**	Fwd: 5'-CAGGTTTTTAAATTTTTCC-3'<br>Rev: 5'-AGGCAGGGTCTCACTAAG -3'
**173 bp (-1142~ -969)**	Fwd: 5'-CCCTGCCTGTCTCTACAA-3'<br>Rev: 5'-ACAGCATCTCACTCTGTCACCTAG-3'
**199 bp (-1000~ -801)**	Fwd: 5'-ACTCCAGCCTAGGTGACAGAG-3'<br>Rev: 5'-CTGTGCTCCCGTAGAATAATTCCC-3'
**Non-specific coding region (1 ~149)**	Fwd: 5'-CTCCAACTGCTGAGGAACCG-3'<br>Rev: 5'-GACAGTCGGAGCTGTAGTCGT-3'
**p53-binding consensus (wild-type)**	Fwd: 5'-TACAGAACATGTCTAAGCATGCTGGGG-3'<br>Rev: 3'-ATG TCTTGTACAGATTCGTACGACCCC-5'
**p53-binding consensus (mutant)**	Fwd: 5'-TACAGAATCGCTCTAAGCATGCTGGGG-3'<br>Rev: 3'-ATGTCTTAGCGAGATTCGTACGACCCC-5'
**p53 promoter (-420~ -84)**	Fwd: 5'-TCTGGGAGAAAACGTTAGGG -3'<br>Rev: 5'-AAGCTGGACAGTCGCCATGA -3'
